# The evolution of genome-scale models of cancer metabolism

**DOI:** 10.3389/fphys.2013.00237

**Published:** 2013-09-03

**Authors:** Nathan E. Lewis, Alyaa M. Abdel-Haleem

**Affiliations:** ^1^Department of Biology, Brigham Young UniversityProvo, UT, USA; ^2^Department of Biology, The American University in CairoNew Cairo, Egypt

**Keywords:** cancer, metabolism, omics, systems biology, constraint-based modeling, data analysis, modeling and simulation, warburg effect

## Abstract

The importance of metabolism in cancer is becoming increasingly apparent with the identification of metabolic enzyme mutations and the growing awareness of the influence of metabolism on signaling, epigenetic markers, and transcription. However, the complexity of these processes has challenged our ability to make sense of the metabolic changes in cancer. Fortunately, constraint-based modeling, a systems biology approach, now enables one to study the entirety of cancer metabolism and simulate basic phenotypes. With the newness of this field, there has been a rapid evolution of both the scope of these models and their applications. Here we review the various constraint-based models built for cancer metabolism and how their predictions are shedding new light on basic cancer phenotypes, elucidating pathway differences between tumors, and dicovering putative anti-cancer targets. As the field continues to evolve, the scope of these genome-scale cancer models must expand beyond central metabolism to address questions related to the diverse processes contributing to tumor development and metastasis.

## Introduction

“One of the goals of cancer research is to ascertain the mechanisms of cancer.” These words, penned by Dulbecco (1986), began a treatise on how a mechanistic understanding of cancer requires a sequenced human genome. Now with the abundance of sequence data, we are finding diverse genetic changes among different cancers (Vogelstein et al., 2013). While we are cataloging these mutations, the associated mechanisms leading to phenotypic changes are often unclear since mutations occur in the context of complex biological networks. For example, mutations to isocitrate dehydrogenase lead to oncometabolite synthesis, which alters DNA methylation and ultimately changes gene expression and the balance of normal cell processes (Sasaki et al., 2012). Furthermore, many different combinations of mutations can lead to cancer. Since the genetic heterogeneity between tumors can be large, the biomolecular mechanisms underlying tumor physiology can vary substantially. This is apparent in metabolism, where tumors can differ in serine metabolism dependence (Possemato et al., 2011) or TCA cycle function (Frezza et al., 2011b). In addition, diverse mutations can alter NADPH synthesis by differentially regulating signaling pathways, such as the AMPK pathway (Cairns et al., 2011; Jeon et al., 2012).

The challenges regarding complexity and heterogeneity in cancer metabolism are beginning to be addressed with the COnstraint-Based Reconstruction and Analysis (COBRA) approach (Hernández Patiño et al., 2012; Sharma and König, 2013), an emerging field in systems biology. Specifically, it accounts for the complexity of the perturbed biochemical processes by using genome-scale metabolic network reconstructions (Duarte et al., 2007; Ma et al., 2007; Thiele et al., 2013). In a reconstruction, the stoichiometric chemical reactions in a cell are carefully annotated and stitched together into a large network, often containing thousands of reactions. Genes and enzymes associated with each reaction are also delineated. The networks are converted into computational models and analyzed using many algorithms (Lewis et al., 2012). COBRA approaches are also beginning to address heterogeneity in cancer by integrating experimental data with the reconstructions (Blazier and Papin, 2012; Hyduke et al., 2013) to tailor the models to the unique gene expression profiles of general cancer tissue, and even individual cell lines and tumors. Here we describe the recent conceptual evolution that has occurred for constraint-based cancer modeling.

## Evolution of model scope and specificity

**Figure 1:**
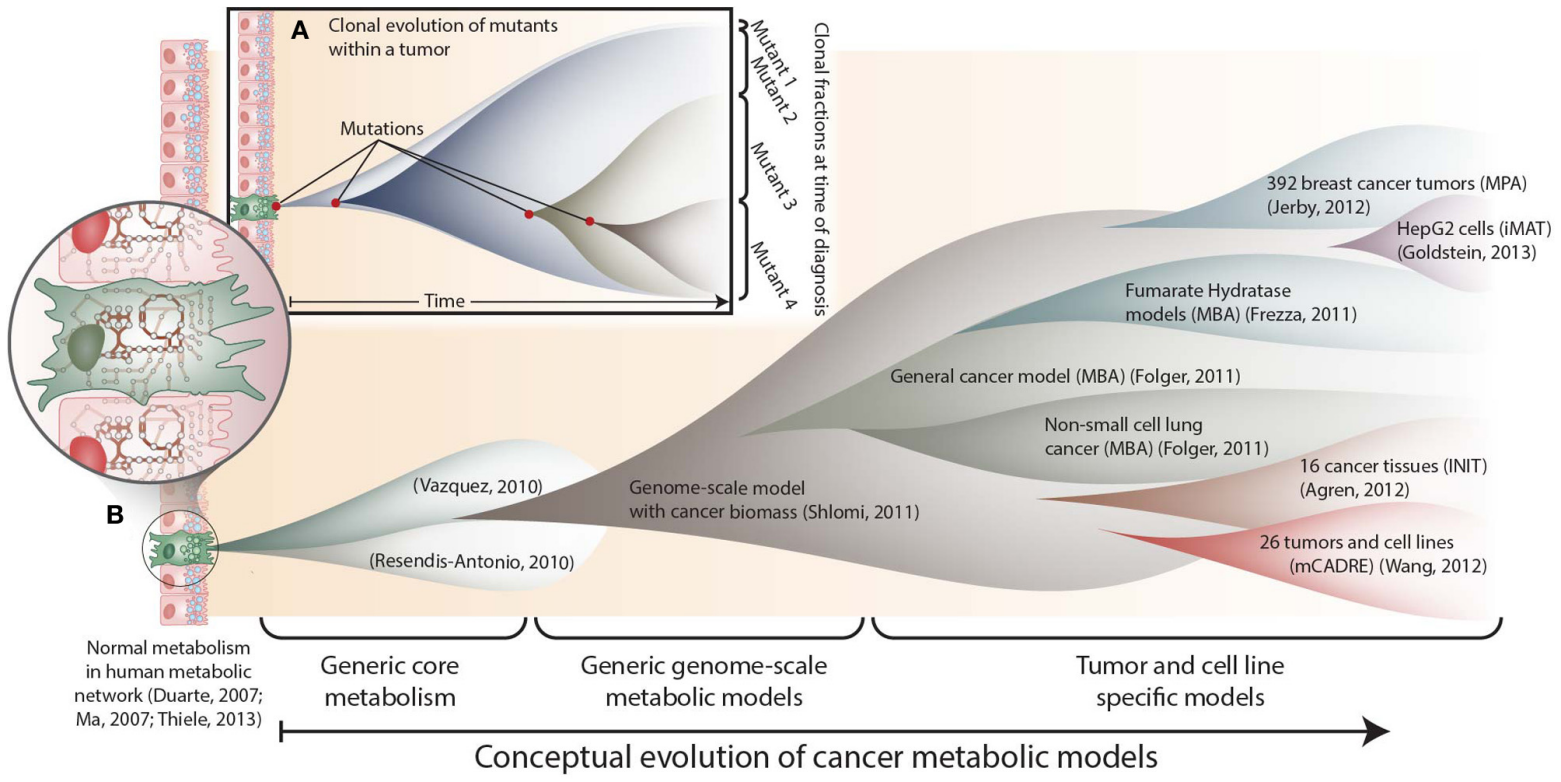
**The conceptual evolution of constraint-based models of cancer metabolism. (A)** Clonal evolution commonly occurs in a developing tumor as new mutations are acquired that confer increased growth capabilities to new mutants. At the time of diagnosis, a tumor often consists of a mixed population of cancerous cells. **(B)** Similarly, the scope and specificity of cancer metabolic models have rapidly evolved over the past few years. Genome-scale metabolic network reconstructions have provided a valuable resource, since they contain thousands of known human metabolic reactions (Duarte et al., 2007; Ma et al., 2007; Thiele et al., 2013). This knowledge enabled the first two cancer-specific metabolic models, which focused on core metabolic pathways(Resendis-Antonio et al., 2010; Vazquez et al., 2010). In 2011, the first genome scale model of general cancer metabolism was used to provide insights into the Warburg effect(Shlomi et al., 2011). Shortly thereafter, transcriptomic data from the NCI-60 cell lines were used to build a general genome-scale model of cancer metabolism, which was used to assess metabolic drug targets(Folger et al., 2011). Now, numerous additional models have been built using data from specific cell lines and tumors. These models have elucidated pathways that differ between tumors(Agren et al., 2012; Wang et al., 2012), identified pathways that are differentially regulated with changes in estrogen receptor or p53 expression(Jerby et al., 2012; Goldstein et al., 2013), and predicted potential anti-cancer drug targets(Folger et al., 2011; Frezza et al., 2011b).

The molecular basis of cancer includes mutations, epigenetic changes, mRNA splice variants, fluctuations in protein expression, etc. Each molecular change influences other cell components, and the perturbed molecular interactions ultimately induce cancer phenotypes. Thus, cancer is a phenotypic manifestation of a dysfunctional biomolecular network. To understand how the complex and heterogeneous changes in cell networks lead to cancer phenotypes, several studies have recently constructed constraint-based metabolic network models of the disease. With each publication, these models have evolved in scope and detail (Figure 1). That is, the first few models represented the coarse-grained canonical commonalities of cancer metabolism, while the more recent models have been specific to individual cell lines, tissues, or patients. Here we compare these models, and discuss the scope of insights they provided.

## Generic cancer models

Cancer is highly complex for two reasons. First, molecular changes occur in the context of a vast network of interactions. Thus, a mutation's impact is not apparent without accounting for the functions of many downstream molecules. Second, the induction of tumorigenesis from one mutation is rare. Multiple mutations accumulate over time as the tumor evolves (Yates and Campbell, 2012) (Figure 1A). The complex context in which cancer mutations reside often confounds efforts to understand their phenotypic link. However, the complexity of cellular networks can be addressed computationally.

Several metabolic properties are common among tumors (Kroemer and Pouyssegur, 2008), such as anaerobic glycolysis, ATP production and growth. Thus, generic models of cancer metabolism were constructed by including major pathways producing ATP or biomass. This was done for two small-scale models of core metabolism (Resendis-Antonio et al., 2010; Vazquez et al., 2010) and another genome-scale metabolic model (Shlomi et al., 2011). Resendis-Antonio et al. (2010) analyzed core metabolic pathways in cancer: glycolysis, TCA cycle, pentose phosphate, glutaminolysis and oxidative phosphorylation. This model accurately predicted HeLa cell line growth rates and identified known drug targets, including lactate dehydrogenase and pyruvate dehydrogenase. The model also recapitulated the Warburg effect, i.e., at a fixed glucose uptake rate, a decrease in pyruvate dehydrogenase flux increased biomass production capacity. Using another model of ATP production, Vazquez and colleagues demonstrated that the Warburg effect may result from molecular crowding in proliferating cells (Vazquez et al., 2010; Vazquez and Oltvai, 2011). This finding was further supported by Shlomi et al. (2011), using a generic genome scale model of human metabolism that was modified to simulate biomass precursor formation as a proxy for cancer cell proliferation.

The initial three constraint-based cancer metabolic models successfully recapitulated general features of cancer metabolism and provided systems-level insights into the Warburg effect. More detailed predictions were obtained from a fourth generic cancer model (Folger et al., 2011). This model was built by mapping transcriptomic data to the human metabolic network (Duarte et al., 2007). Using the Model Building Algorithm (MBA; Jerby et al., 2010) to remove pathways that were not supported by the data, a model with 772 reactions and 683 genes was obtained. The MBA general cancer model was built as follows. Highly expressed genes in the NCI-60 cell lines were identified from transcriptomic data. Reactions associated with the highly expressed genes were called “core” reactions and assumed to be active in cancer cells. Then non-core reactions were removed if all core reactions remained functional. The model included a biomass objective function to simulate the synthesis of all metaboltes needed for cell proliferation (e.g., nucleotides, amino acids, lipids, etc.). Thus, this model was the first genome-scale model of cancer metabolism that captured the main metabolic functions shared by many cancer types, while removing pathways that were not endemic to cancer.

Flux Balance Analysis (FBA) (Orth et al., 2010) was used with the generic MBA cancer model to identify potential drug targets (Folger et al., 2011). Since FBA simulates a cell's metabolic state (including growth and metabolic flux), the phenotypic response following gene knockdowns can be predicted, and the effects of drug applications can be simulated on a large scale. In the MBA cancer model, genes were identified that, when inhibited, decreased the model-predicted growth rate. In doing so, 52 cytostatic drug targets were identified, of which 40% are targeted by known, approved, or experimental anticancer drugs. Predictions were also made for pairs of synthetic lethal drug targets. The synergistic effects of these target pairs were validated by looking for increases in drug susceptibility among NCI-60 cell lines lacking expression of one target in each synthetic lethal pair. Cell lines missing one target were often more susceptible to treatments against the other gene in the synthetic lethal pair. Thus, by accounting for cancer-specific combinations of metabolic pathways, one could design therapeutics to inhibit cancer cell proliferation.

## Cell line and tumor-specific cancer models

General cancer models have demonstrated that COBRA approaches can manage the complexity of cancer metabolism. While there are common biochemical features and mutations, there remains much heterogeneity between different tumors. Thus, algorithms are now generating genome-scale metabolic models specific to cancer cell lines and tumors. These models provide varying levels of insight by elucidating cancer-specific pathways and predicting therapeutic targets for specific tumors.

### Identifying cancer-relevant pathways

Two studies recently identified metabolic pathways that differ between cancer and the parent tissue from which the tumor arose (Agren et al., 2012; Wang et al., 2012). To do this, algorithms were developed to integrate omic data with a reference human metabolic reconstruction to build tumor-specific metabolic networks.

One algorithm, called Integrative Network Inference for Tissues (INIT), used immunohistochemical staining data (Uhlen et al., 2010), metabolomic data (Wishart et al., 2013), and transcriptomic data to construct metabolic models for 16 cancer types and their parent tissues (Agren et al., 2012). These models each contained more than 2600 reactions. Genes and reactions that were more frequently identified in the cancer tissues were analyzed to identify Reporter Metabolites (Patil and Nielsen, 2005) that were more frequently associated with cancer. Several metabolites arose as dominant features in cancer. These included polyamines (e.g., spermine, spermidine, and putrescine), intermediates of isoprenoid biosynthesis (e.g., geranylgeranyl diphosphate), prostaglandins and leukotrienes. Previous studies targeted these processes in cancer, but this study predicted key sites where the pathways could be targeted. In addition, bilirubin and biliverdin arose as novel targets. By targeting biliverdin reductase, one could potentially block the anti-oxidative stress functions of these metabolites, thus enhancing cell death.

Another 26 tumor-specific genome-scale models were generated using an algorithm called Metabolic Context-specificity Assessed by Deterministic Reaction Evaluation (mCADRE) (Wang et al., 2012), and the sizes of these models ranged from roughly 1000–1400 reactions. Each cancer model was compared to a corresponding healthy tissue model. Cancer-specific pathways were identified, many of which were previously known to contribute to tumorigenesis and neoplastic growth, including folate metabolism, eicosanoid metabolism, and nucleotide metabolism. For example, folate and nucleotide metabolism have previously been chemotherapy targets since they contribute to the increased nucleotide synthesis rate in cancer. Beyond general pathway differences, several reactions were identified as being more frequently associated with cancer models. These included eicosanoid metabolism reactions catalyzed by 5-lipoxygenase, which contributes to angiogenesis and proliferation (Ye et al., 2005). Importantly, the association of these metabolic pathways with cancer was not apparent when the authors only looked at differential gene expression without the model topology. Thus, it is clearly advantageous to study gene regulatory programs in cancer in the context of functional metabolic networks.

### Assessing the metabolic phenotype of tumors

Recently, models were built to elucidate p53′s role in regulating cancer metabolism (Goldstein et al., 2013). This was accomplished using an algorithm called integrative Metabolic Analysis Tool (iMAT) (Shlomi et al., 2008), which uses gene expression levels to predict the distribution of metabolic fluxes. iMAT uses an optimization problem to maximize the number of highly expressed genes that carry flux while minimizing the number of low expression genes that must be used. Using iMAT, models were constructed for cell lines with different levels of p53 expression. Specifically, two liver-derived HepG2 cell lines were developed, expressing either a short hairpin RNA (shRNA) targeting p53 or a control shRNA. The cell lines were grown with or without Nutlin-3a, which activates p53, and then the transcriptomes of the samples were assayed. For each condition, iMAT models were constructed, and these models demonstrated that p53 increases the expression of gluconeogenesis. Thus, p53 may be diverting glucose away from growth-promoting pathways, such as glycolysis and the pentose phosphate pathway, thereby inhibiting neoplastic growth and tumorigenesis.

Another method, called Metabolic Phenotype Analysis (MPA), elucidated metabolic features among 392 breast cancer tumors, based on microarray data (Jerby et al., 2012). To do this, several cellular metabolic functions were defined (e.g., lipid production). Then, for each breast cancer sample, microarray data were used to constrain the human metabolic model using a variant of iMAT. For each metabolic function, a score was assigned to describe the fitness of the given tumor sample for performing the metabolic function of interest. Across all samples, the authors compared metabolic functions, growth rates, posttranscriptional regulation, and metabolic biomarkers. For example, the authors found that premalignant cells grow faster than malignant tumors, suggesting a proliferative deceleration prior to metastasis. In addition, late-stage tumors showed increased flux in glycolysis, lactate production, ROS detoxification, and the pentose phosphate pathway.

MPA further elucidated metabolic differences between tumors differing in their estrogen receptor (ER) status (commonly used to differentiate between breast cancer types) (Jerby et al., 2012). Specifically, between ER^+^ and ER^−^ tumors, 73% of the metabolic processes had significantly different MPA scores. For example, glutamine biosynthesis/secretion and lactate production were more pronounced in ER^+^, while serine metabolism and glutamine uptake were more predominant in ER^−^. These differences resulted from a stoichiometric tradeoff between glutamine secretion and serine metabolism, consistent with the observation that serine biosynthesis requires glutamine as a nitrogen donor (Possemato et al., 2011). Thus, the use of models for -omic data analysis can elucidate the biomolecular mechanisms underlying complex phenotypes in different tumors.

### Model-guided inhibition of cancer cell proliferation

The biochemical detail provided by COBRA models is elucidating differences between tumors. Can the models also predict therapeutic targets? While the constraint-based cancer modeling field is young, two studies demonstrated the predictive power of cell line-specific models. Folger et al. (2011) built a cancer model for non-small cell lung cancer metabolism, based on microarray data. Model-predicted genes that were essential for growth significantly overlapped with experimentally measured essential genes from an shRNA screen. Furthermore, the overlap was more significant than when the test was repeated with the general cancer MBA model, thus demonstrating that cell-line specific models could suggest novel targets.

A subsequent study found and validated a target relevant to hereditary leiomyomatosis and renal cell cancer (HLRCC). This cancer can develop when the tumor suppressor gene fumarate hydratase (FH) is mutated. To mimic HLRCC, a murine renal cell line was derived, and subsequently FH was disabled (Frezza et al., 2011b). MBA models were constructed for the cell line before and after disabling FH. Simulations with these models demonstrated that a loss of FH is buffered by other pathways. Indeed, 24 model genes were synthetic lethal with FH, most of which contributed to heme biosynthesis. When an inhibitor for heme oxygenase was used, FH-deficient cells could not proliferate. Since normal cells are FH^+^, they were relatively unaffected by the therapeutic inhibition of heme oxygenase. Thus, a cell-line specific model enabled the identification of a potential new drug target for a specific tumor type, highlighting the potential of model-predicted cancer therapies.

## Expanding the scope of genome-scale models in cancer

Insight into cancer metabolism may be expanded as genome-scale metabolic network models are further analyzed using the expanding toolbox of constraint-based modeling methods (Price et al., 2004; Lewis et al., 2012). A current challenge, however, is to increase the scope of genome-scale cancer modeling. Three directions of relevance to cancer include: (1) employing models as data integration platforms, (2) using models to discover details about mutations and enzyme regulation, and (3) expanding models to account for the other hallmarks of cancer.

True to Dulbecco's vision (Dulbecco, 1986), the human genome sequence enables systematic approaches and novel technologies to understand cancer. Numerous cancer genomes have been sequenced (Hudson et al., 2010), and their mutations are being cataloged (Forbes et al., 2011). Others have profiled cancer chromatin landscapes (Schuster-Böckler and Lehner, 2012) and studied dysregulated transcriptional regulatory programs in cancer (Lee et al., 2012; Lee and Young, 2013). Protein modifications are routinely identified and proteins are being quantified (Cohen et al., 2008; Uhlen et al., 2010). In parallel, other studies are characterizing the metabolome (Jain et al., 2012) and metabolic flux through pathways in cancer (Wellen et al., 2010; Locasale et al., 2011).

**Figure 2:**
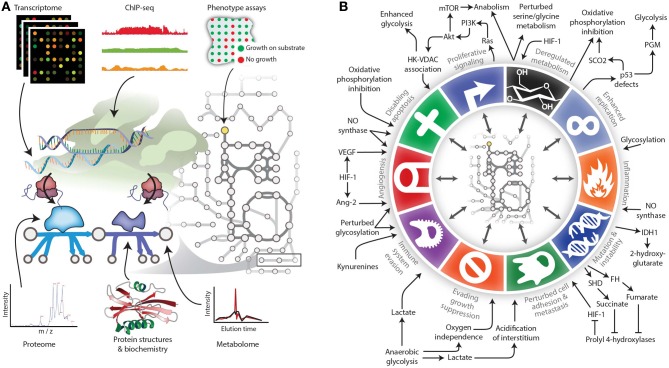
**Expanding the reach of genome-scale metabolic models for studying cancer. (A)** Genome-scale metabolic models serve as biochemically-supported data-integration platforms. In the metabolic network, metabolomic data can be associated with metabolites, while genomic, transcriptomic, proteomic, and related data types can be associated with metabolic reactions. Phenotypic measurements can be used to constrain properties of the network such as growth rate under certain experimental conditions. **(B)** The various Hallmarks of Cancer either affect metabolism or are modulated by metabolic changes. Therefore, modeling techniques are needed to account for these interactions between metabolism and other cell processes. Panel B adapted from (Hanahan and Weinberg, 2011) and (Kroemer and Pouyssegur, 2008) with permission.

Genomic, metabolomic, and phenotyping studies yield valuable data, but it is challenging to integrate the datasets for deeper insight. Relationships between disparate data types can be unclear. Some successes have relied on complex statistical methods to find loci that co-vary with metabolites (Kettunen et al., 2012; Krumsiek et al., 2012). Genome-scale metabolic models provide a complementary approach. Their mechanistic context with physical interaction data enables one to integrate metabolomic, transcriptomic, proteomic, genomic, and high-throughput phenotyping array data (Figure 2A) (Blazier and Papin, 2012; Hyduke et al., 2013). For example, changes in the extracellular metabolome can be used to constrain model uptake and secretion fluxes. Then transcriptomic and proteomic data can constrain internal fluxes, and phenotypic assays provide limits on growth rates or other phenotypic measures. Thus, these models become biochemically-based data integration platforms. This will likely become increasingly common for interpreting cancer omic data.

Metabolism significantly changes during tumorigenesis and metastasis (Cairns et al., 2011). This stems from numerous biochemical adjustments, including mutations and posttranslational modifications (PTMs) (Solit and Mellinghoff, 2010). Ongoing studies biochemically characterize how aberrant PTMs and mutations regulate metabolism in cancer, but the low-throughput nature of biochemistry prohibits the characterization of all mutations. Fortunately, emerging approaches are beginning to prioritize and infer the functional impact of biomolecular changes (Ng and Henikoff, 2006; Beltrao et al., 2012). Furthermore, developments in constraint-based modeling and metabolic flux analysis are beginning to predict regulatory roles of PTMs in metabolism (Oliveira et al., 2012). As cancer models further improve, they will likely help to rapidly characterize biochemical changes in cancer.

Several mutations have been repeatedly witnessed in metabolic enzymes in tumors. Many of these changes also influence other Hallmarks of Cancer (Kroemer and Pouyssegur, 2008; Hanahan and Weinberg, 2011) (Figure 2B). For example, mutations in succinate dehydrogenase and fumarate hydratase eventually lead to the activation of the HIF-1 transcription factor. HIF-1 regulates invasion, cell survival, angiogenesis, and inflammation in cancer (Semenza, 2003; Frezza et al., 2011a). Conversely, some changes in metabolic flux are downstream effects of perturbed transcriptional regulatory and signaling systems. For example, p53 expression influences gluconeogenesis (Goldstein et al., 2013), and mTOR signaling influences pyrimidine synthesis (Robitaille et al., 2013). Furthermore, metabolic changes in cancer influence cell-cell interactions in tumors. For example, changes in kynurenine concentration induce immune suppression, thereby facilitating immune escape in cancer (Prendergast, 2008). Metabolic exchanges also occur between cancer cells and the surrounding stroma (Giatromanolaki et al., 2012). These are just a few examples of diverse cell processes that interact with metabolism throughout the stages of cancer.

Advances in constraint-based modeling are now addressing cell–cell interactions(Lewis et al., 2010; Bordbar et al., 2011; Zomorrodi and Maranas, 2012; Levy and Borenstein, 2013) and incorporating other cellular processes into these models, including transcription regulation, translation, and signaling (Lee et al., 2008; Karr et al., 2012; Lerman et al., 2012; Thiele et al., 2012, 2010; Simeonidis et al., 2013). Efforts to link these processes to cancer metabolism should be fruitful. Lastly, models are needed that incorporate the hallmark processes of cancer, such as angiogenesis, apoptosis evasion, and cell adhesion. Such models will allow COBRA methods to reach beyond metabolism for a more holistic view of dysregulated processes in cancer.

## Conclusion

When Renato Dulbecco pleaded with the scientific community to sequence the entire human genome (Dulbecco, 1986), seeds were sown for the genomic era of cancer biology. Now we are witnessing early developments in genome-scale modeling of cancer metabolism. The recent modeling successes serve as a harbinger to the discoveries that, in conjunction with advances in experimental tools, will deepen our understanding of cancer biology and our success in treating diverse classes of cancer.

### Conflict of interest statement

The authors declare that the research was conducted in the absence of any commercial or financial relationships that could be construed as a potential conflict of interest.
